# Supplementary figures and images for: Pan-cancer landscape of protein kinase D3: An integrative TCGA multi-omics analysis of clinical, molecular, and immunological roles
Source: PLoS One. 2026 Apr 3;21(4):e0346173. doi: 10.1371/journal.pone.0346173 (PMC13048501; doi:10.1371/journal.pone.0346173)

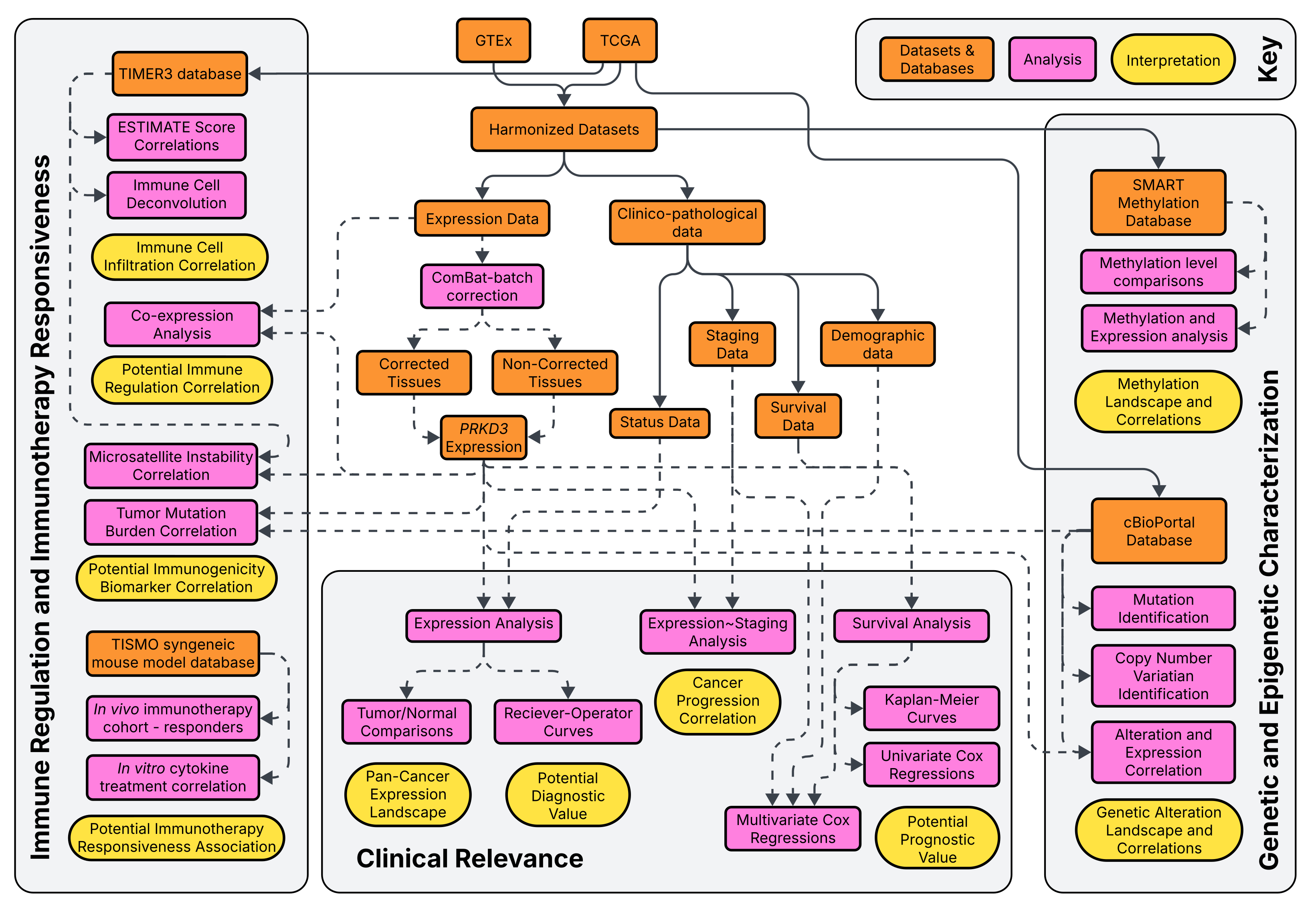

Supplement: S1 Fig — Schematic overview of the integrated analytical pipeline used to characterize PRKD3 across cancers. (TIFF) [file pone.0346173.s001.tiff]

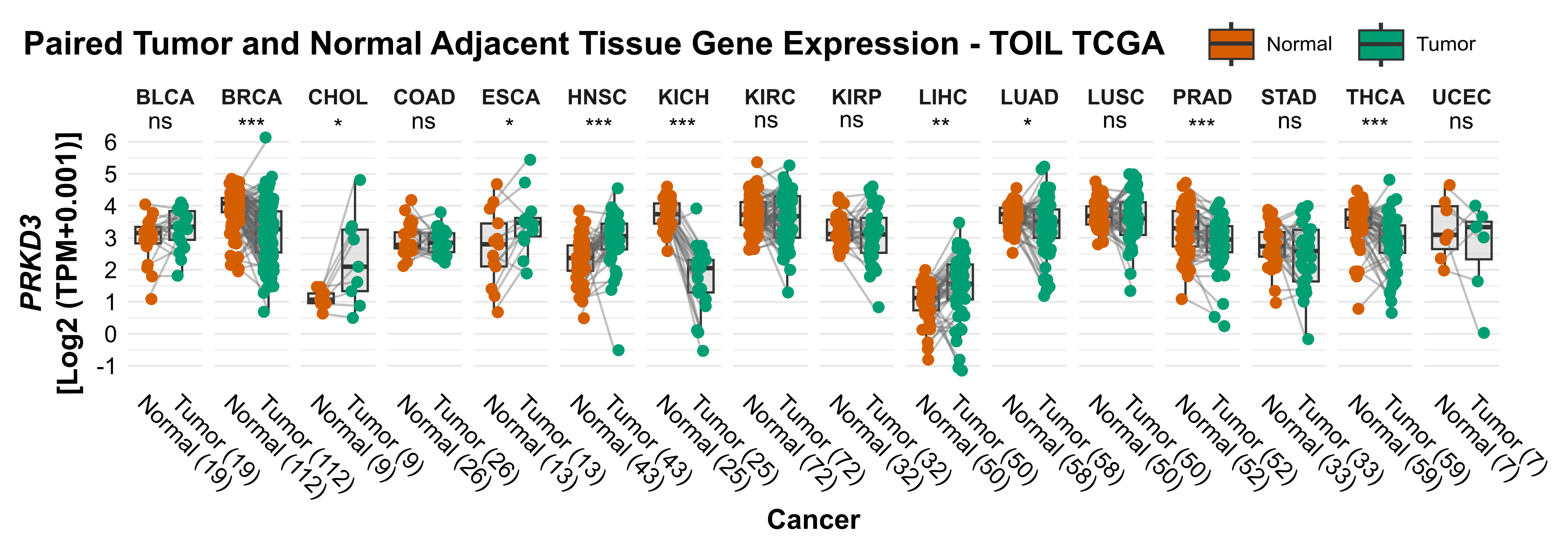

Supplement: S2 Fig — Statistical significance was assessed using the Wilcoxon test and is indicated as follows: *FDR < 0.05, **FDR < 0.01, ***FDR < 0.001. (TIFF) [file pone.0346173.s002.tiff]

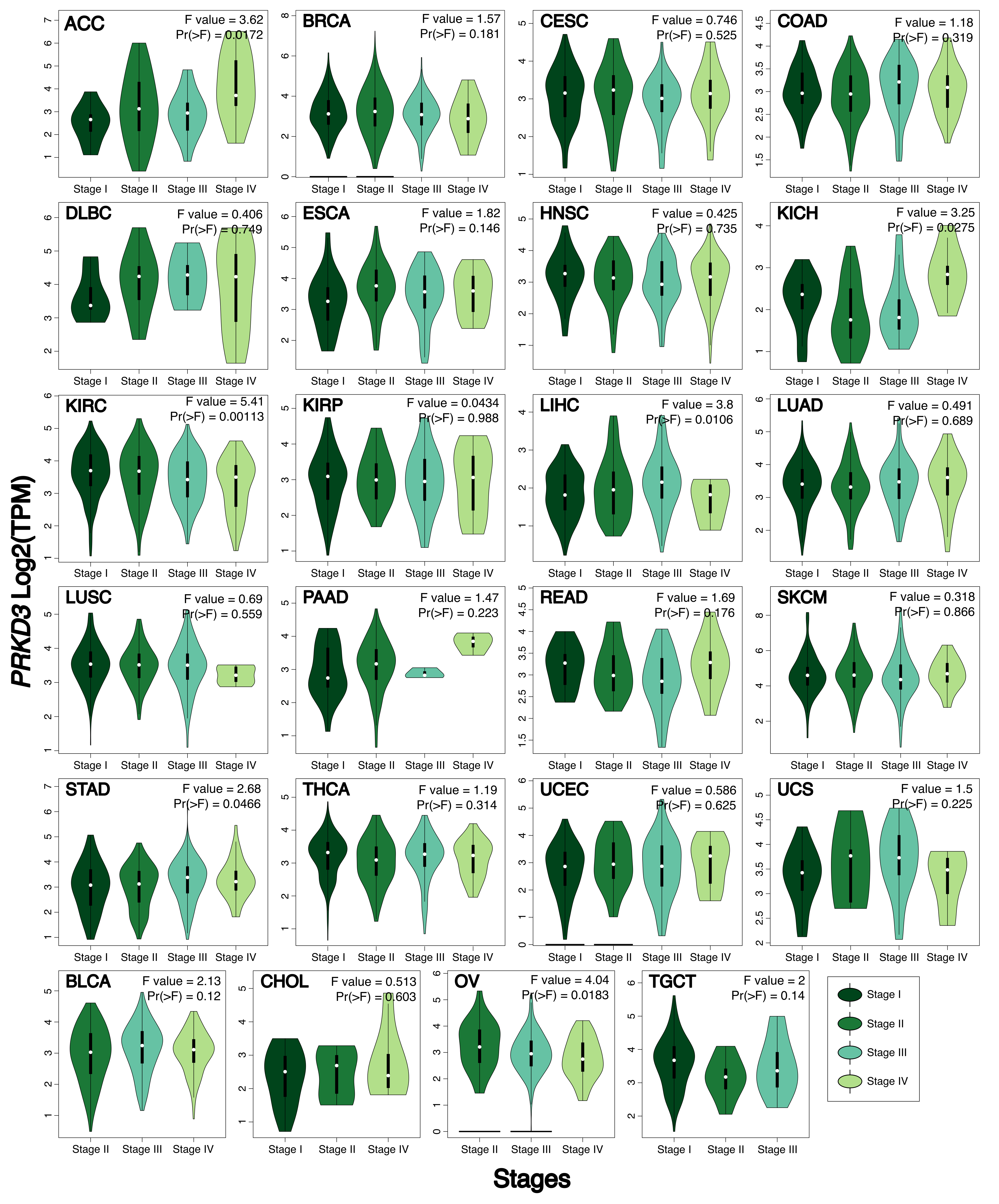

Supplement: S3 Fig — Different shades of green represent distinct cancer stages. Data were obtained from the GEPIA2.0 platform. (TIFF) [file pone.0346173.s003.tiff]

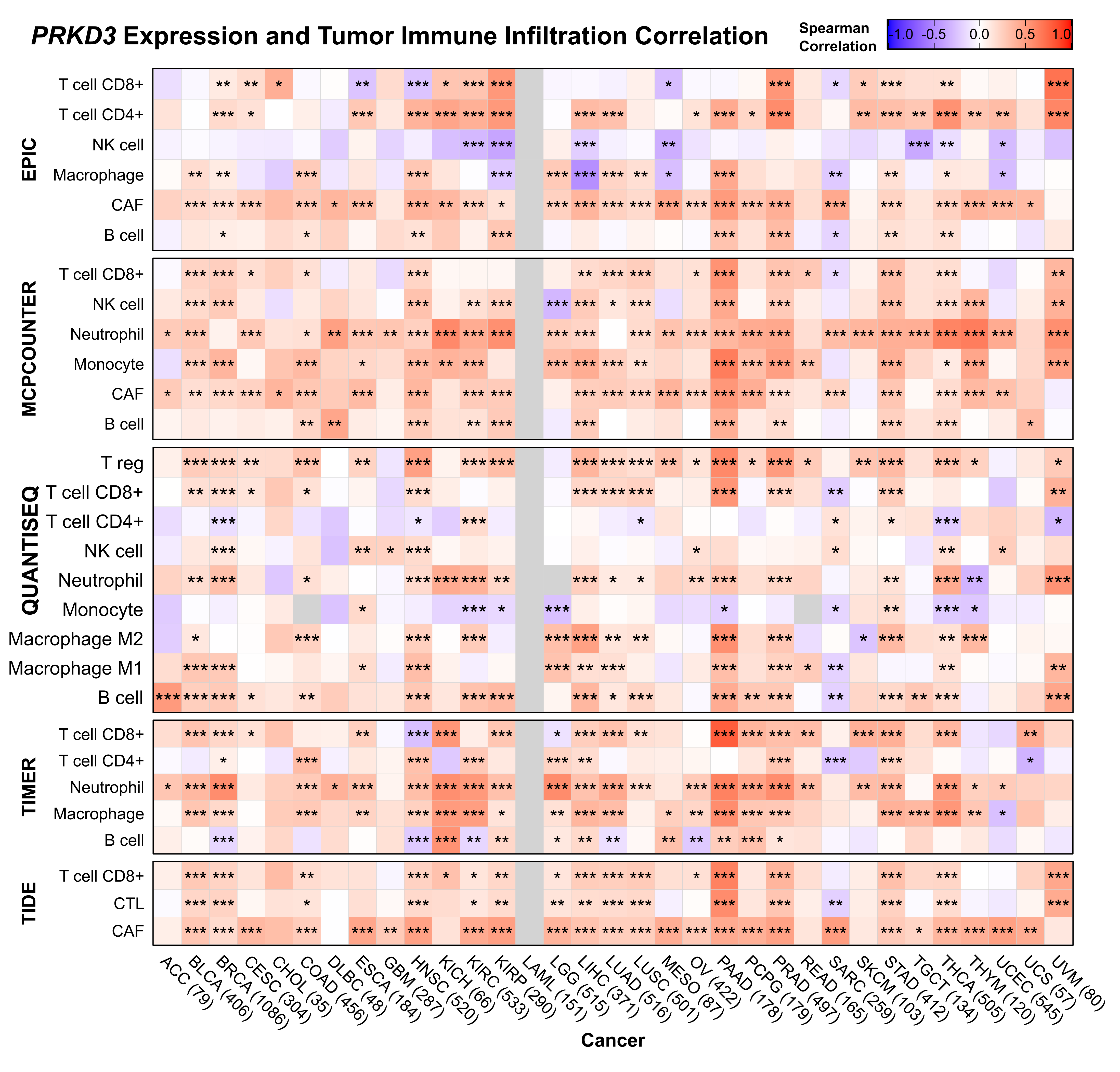

Supplement: S4 Fig — Box color represents Spearman correlation coefficients, with red indicating positive correlations and blue indicating negative correlations. Statistical significance is indicated as: *FDR < 0.05, **FDR < 0.01, ***FDR < 0.001. (TIFF) [file pone.0346173.s004.tiff]
