# Supplementary material for: Pan-cancer landscape of protein kinase D3: An integrative TCGA multi-omics analysis of clinical, molecular, and immunological roles
Source: PLoS One. 2026 Apr 3;21(4):e0346173. doi: 10.1371/journal.pone.0346173 (PMC13048501; doi:10.1371/journal.pone.0346173)
Supplement: Attachment — Submitted filename: Response to Reviewer N2.docx [file pone.0346173.s009.docx]

**Response to Reviewer #2 Comments**

We would like to thank the reviewer for taking the time to review this manuscript. Please find the detailed responses below and the corresponding revisions.

**Reviewer #2: This manuscript presents a comprehensive pan-cancer analysis of PRKD3 using multi-omics datasets from TCGA, GTEx, CPTAC, cBioPortal, and multiple immune-deconvolution platforms. The study examines PRKD3 expression, genetic and epigenetic alterations, clinicopathological associations, prognostic value, immune infiltration patterns, and potential relevance to immunotherapy. Overall, the work addresses an important gap by providing a broad, data-driven overview of PRKD3 across 33 cancer types and offers useful descriptive insights that may inform future mechanistic studies. However, several methodological, statistical, and interpretational issues need to be addressed to strengthen the rigor and reliability of the conclusions.**

**Introduction**

1. **The introduction provides a thorough overview of the kinase family and successfully contextualizes PRKD3 as a biologically relevant but understudied gene in cancer. However, the background is somewhat lengthy and occasionally repetitive. The authors may tighten the narrative and present clearer, more specific hypotheses or objectives to guide the reader. While the need for a pan-cancer investigation is justified, stating explicit research questions or expected outcomes would enhance clarity.**

We thank the reviewer for this feedback. In response, we have synthesized the Introduction section and eliminated redundancies while retaining essential context on cancer statistics, PKD family background and *PRKD3's* understudied oncogenic roles. We have also restructured a final paragraph that indicates the research objectives that guide our study:

In line 57: *“In this study, we conducted a comprehensive pan-cancer analysis of PRKD3 using integrated TCGA, GTEx, and multi-omics datasets to characterize its expression patterns across tumor and normal tissues, evaluate associations with patient survival and tumor progression, and investigate genetic/epigenetic alterations with implications for immunoregulation and therapy response. We further assessed PRKD3 relationships with tumor immune microenvironment, including stromal/immune cell infiltration and immune modulators, as well as predictors of immune checkpoint inhibitor efficacy such as tumor mutation burden (TMB) and microsatellite instability (MSI). Finally, we explored the potential relationship between PRKD3 and the therapy efficiency in different immunotherapy syngeneic models.”*

**Methods**

**The study makes use of multiple public resources, which is appropriate for a pan-cancer bioinformatics analysis. However, several methodological gaps reduce the technical rigor:**

1. **Direct comparison between TCGA tumors and GTEx normal samples is performed without any batch-effect correction. This is a well-known issue and can significantly bias differential expression results and diagnostic ROC analyses.**

We thank the reviewer for highlighting this critical methodological concern. Following the established pan-cancer RNA-seq data harmonization procedures by Wang et al. (2018), we applied ComBat batch correction to the TCGA-GTEx TOIL TPM data, removing dataset-specific technical effects while preserving biological variation between tumor and normal TCGA/GTEx samples. All differential expression analyses and ROC curves now explicitly indicate batch correction status per cancer type in the revised Methods (lines 93-95) and Results (Figures 1, 3, and S1).

Wang Q, Armenia J, Zhang C, Penson A V., Reznik E, Zhang L, et al. Unifying cancer and normal RNA sequencing data from different sources. Sci Data. 2018;5. doi:10.1038/sdata.2018.61

2. **Prognostic analyses do not adjust for essential confounders such as stage, age, tumor purity, and sex. Multivariate Cox regression is required to determine whether PRKD3 is an independent prognostic factor.**

We thank the reviewer for emphasizing the importance of multivariate adjustment. In addition to univariate Cox regressions, we conducted comprehensive multivariate analyses adjusting for age, gender, race, and pathological stage. These results, now detailed in the revised Results section and Supplementary Table S4, confirm *PRKD3* as an independent prognostic factor in multiple cancers:

*"Multivariate Cox regressions adjusting for age, gender, race, pathological stage, and tumor purity showed independent OS associations with elevated HR for high PRKD3 expression in ACC (HR=4.04, p=0.002), KICH (HR=14.7, p=0.01), LGG (HR=1.61, p=0.03), LIHC (HR=1.54, p=0.02), and STAD (HR=1.45, p=0.04), but reduced HR in KIRC (HR=0.71, p=0.02) and LUAD (HR=0.71, p=0.03). Multivariate PFI analyses confirmed elevated HR in ACC (HR=3.91, p=0.003) and LGG (HR=1.25, p=0.04)."*

These findings demonstrate *PRKD3's* cancer-specific independent prognostic value. All methodological details and full results tables with p-values/confidence intervals are provided in the updated Supplementary Table S4.

3. **TIMER, MCP-counter, EPIC, TIDE, and QuanTIseq often produce discordant cell-type estimates. The manuscript reports findings but does not explain how inconsistencies were handled or which algorithmic outputs were prioritized.**

We appreciate the reviewer raising this issue regarding immune deconvolution discordance. To identify consensus immune infiltrate associations with *PRKD3*, we performed a meta-analysis of Spearman correlation coefficients (ρ) across the five methods (TIMER, MCP-counter, EPIC, TIDE, QuanTIseq), requiring agreement from ≥3 algorithms per cancer type. Using Fisher Z-transformation, we calculated pooled ρ estimates and heterogeneity (I² statistic), as detailed in revised Methods and Results (lines 129 and 321). Individual method correlations remain in Supplementary Figure S3. This approach addresses handles discordance while prioritizing consensus findings.

4. **The manuscript states that FDR correction was applied, but this is not clearly indicated for all analyses. Some figures appear to show uncorrected p-values. Please specify the exact correction method (e.g., Benjamini–Hochberg) and where it was applied.**

We thank the reviewer for indicating this clarification. All differential expression analyses (Wilcoxon rank-sum tests) and Spearman correlations underwent Benjamini-Hochberg FDR correction via R. We added a dedicated *"Statistical Analysis"* subsection in Methods (lines 178-185):

*"R (v4.3.3) and associated packages were used for all statistical analyses. Differential expression comparisons employed Wilcoxon rank-sum tests. Clinicopathological, genetic, epigenetic, and immune correlations were assessed via Spearman's rank correlation. Multiple testing correction was performed using the Benjamini-Hochberg false discovery rate (FDR) procedure, with FDR < 0.05 considered statistically significant. Survival analyses used Kaplan-Meier estimation and Cox proportional hazards regression."*

All figure legends now explicitly denote corrected p-values: *“Statistical significance is indicated as *FDR < 0.05, **FDR < 0.01, ***FDR < 0.001.”*

5. **Functional implications of CpG island vs. open sea methylation are not fully discussed. Additionally, methylation–expression correlations should consider CNV as a confounder.**

We thank the reviewer for highlighting these important epigenetic interpretation issues. We discussed the potential functional implications of CpG island vs. open sea methylation.

Line 438: *“Notably, methylation within CpG island probes, putatively overlapping a silencer element, showed inverse correlations with PRKD3 expression in several cancers, consistent with repressive effects on transcription, but a positive association in KIRC. In contrast, N-shore probes, which encompass an enhancer-like region, generally displayed negative methylation–expression correlations, suggesting that hypomethylation at these sites could maintain enhancer activity and support PRKD3 transcription. Open sea sites exhibited more heterogeneous relationships, compatible with context-dependent effects on distal regulatory elements. Together, these data support a model in which site-specific methylation across silencer- and enhancer-enriched regions exerts opposing influences on PRKD3 expression, contributing to its tumor- and context-specific dysregulation.”*

Additionally, we addressed the need for considering CNVs as a potential cofounder in methylation–expression correlations as a limitation in our study.

Line 490: *“Moreover, methylation–expression relationships were not systematically adjusted for copy number variation, which may act as an important confounder in interpreting epigenetic regulation of PRKD3.”*

**Overall, the methods are broadly appropriate, but these omissions weaken the statistical soundness of the study.**

**Results**

**The results are extensive and clearly organized, covering expression, stage correlations, survival, CNV/mutations, DNA methylation, immune infiltration, immune checkpoint associations, and immunotherapy relevance. However, several issues need to be addressed:**

1. **Several conclusions imply causality (e.g., “PRKD3 modulates immune interactions”), although all findings are purely correlative.**

We thank the reviewer for this important caution regarding causal language. All instances implying direct causation have been revised to emphasize correlative associations and potential regulatory roles. Some examples include:

Line 250: *"Overall, our findings highlight the complex role of PRKD3 as both a potential diagnostic and prognostic biomarker across diverse cancer types."*

Line 353: *"Collectively, these findings suggest that PRKD3 expression is intricately linked to multiple layers of immune regulation across cancers... potentially modulating tumor immunogenicity and influencing responsiveness to ICI therapy."*

Line 379: *"...these findings suggest that PRKD3 may influence tumor immune responsiveness through modulation of the tumor microenvironment and immune signaling pathways in syngeneic models."*

Additionally, we reinforced the correlative nature of our findings rather than causal links: “*Overall, our findings propose PRKD3 as a potential context-dependent biomarker to inform precision oncology; however, further validation studies are needed to establish mechanistic roles and clinical utility across diverse tumor types.”*

2. **AUC values from TCGA vs GTEx may be inflated due to uncorrected batch effects and lack of external validation datasets.**

We thank the reviewer for highlighting these critical validation issues. To address potential batch effects inflating AUC values, ComBat correction was applied to TCGA-GTEx TOIL TPM data across cancer cohorts with sufficient normal samples, removing dataset-specific technical effects while preserving biological tumor/normal variation. In the revised Figure 2, we clearly distinguish batch-corrected from non-corrected ROC curves. Full ROC data is available in Supplementary Table S2. Additionally, we acknowledging limitations requiring external clinical validation in our discussion:

Line 417: "*Given limited prior data on PRKD3 clinicopathological associations and expression discrepancies, further clinical studies across diverse patient cohorts and molecular subtypes required to validate the potential links reported in this study."*

3. **TISMO datasets are based on mouse syngeneic models, which differ from human tumors. Their relevance to clinical immunotherapy response should be more carefully qualified.**

We thank the reviewer for emphasizing the translational limitations of mouse models. We have clarified throughout our manuscript that TISMO datasets derive from mouse syngeneic immunotherapy studies and qualified their potential role their clinical relevance in the Discussion. These revisions transparently frame the hypothesis-generating nature of these findings while calling for human validation studies

Line 484: *"Although derived from mouse syngeneic models that differ from human tumors, these data suggest PRKD3 potentially modulates immune responsiveness through microenvironmental and signaling alterations, indicating cancer-specific predictive value as a potential immunotherapy biomarker that warrants further human clinical validation."*

4. **The concept of PRKD3 acting as a “paradox gene” is interesting, but conclusions require analyses that adjust for tumor purity, immune subtypes, or molecular subtypes**.

We appreciate the reviewer’s insightful comments on the “paradox gene” concept. In the revised Discussion, we emphasized its speculative and hypothetic nature and explicitly highlights the need for a more in-depth molecular analysis.

Line 424: *“To our knowledge, PRKD3 has not previously been characterized as a paradoxical gene; hence, we hypothesize a temporal expression dynamic involving initial suppression during tumor initiation followed by intra-tumoral upregulation that drives aggressive progression. These cancer type-specific patterns suggest PRKD3 functions as a potential context-dependent oncogene, with prognostic significance determined by intra-tumoral expression gradients rather than absolute tumor-normal differences. Future studies adjusting for tumor purity, immune subtypes, and molecular classifications will be essential to validate these observations and elucidate the underlying biological mechanisms.”*

**Discussion**

**The discussion synthesizes findings well and connects them to existing literature. The emphasis on context-dependent roles of PRKD3 is appropriate. However, the discussion is overly long and occasionally repetitive. Certain interpretations go beyond what the data support, especially regarding immune regulation and therapy response. The limitations section should explicitly acknowledge the statistical and methodological issues noted above: lack of batch correction, absence of multivariate survival analysis, immune algorithm discrepancies, constraints of mouse-model immunotherapy data. A more concise and conservative discussion will strengthen the manuscript.**

We thank the reviewer for the feedback on the discussion structure and interpretation. (a) Causal language has been systematically revised to emphasize correlative associations and potential regulatory roles throughout (see previous response). (b) Combat batch correction, survival multivariate cox regressions adjusting for age/gender/race/stage and identification of concordant immune infiltrations across methods were performed in this revision to address methodological limitations suggested by the reviewers. (c) Additionally, we further referred to several associated aspects to these limitations in our discussion.

Line 488: *"Our study has several limitations that should be acknowledged. First, all analyses were conducted using publicly available datasets without external cohorts or additional experimental or clinical validation, which limits causal inference and limits the generalizability of our findings. Moreover, methylation–expression relationships were not systematically adjusted for copy number variation, which may act as an important confounder in interpreting epigenetic regulation of PRKD3. The immunotherapy-related analyses also rely on mouse syngeneic models and in vitro systems, which only partially represent human tumor immunobiology and therefore requires cautious extrapolation to clinical contexts."*

**Conclusion**

**The conclusion effectively summarizes key findings, but it should more clearly reflect the descriptive nature of the study and avoid suggesting causal roles for PRKD3. Emphasizing that the results generate hypotheses rather than definitive mechanistic insights would better align with the analyses performed.**

We thank the reviewer for this comment. The revised conclusion now clearly emphasizes the correlative of our comprehensive pan-cancer analyses and addresses our finding as potential roles, while eliminating causal implications:

Line 503: *“In conclusion, our study provides a comprehensive overview of PRKD3 across pan-cancer, suggest a potential role as a context-dependent modulator of tumor biology, prognosis, and immune responsiveness. High expression consistently associated with poor outcomes and increased stromal/ neutrophil infiltration in ACC, LIHC, and STAD, while KIRC exhibited paradoxical associations. PRKD3 also correlated with key immune checkpoints and MSI/TMB status across cancers, alongside cancer-specific patterns in TISMO immunotherapy datasets, highlighting its potential role in shaping the TIME and influencing immunotherapy response. Overall, our findings propose PRKD3 as a potential context-dependent biomarker to inform precision oncology; however, further validation studies are needed to establish mechanistic roles and clinical utility across diverse tumor types.”*

**This manuscript provides a valuable and comprehensive description of PRKD3 across cancers. However, several methodological, statistical, and interpretational issues need to be addressed to ensure technical rigor. The study has the potential to make a meaningful contribution once these concerns are resolved.**
